# Electrical behaviour and evolutionary computation in thin films of bovine brain microtubules

**DOI:** 10.1038/s41598-021-90260-0

**Published:** 2021-05-24

**Authors:** Eléonore Vissol-Gaudin, Chris Pearson, Chris Groves, Dagou A. Zeze, Horacio F. Cantiello, María del Rocio Cantero, Michael C. Petty

**Affiliations:** 1 Department of Engineering, Durham University, South Road, Durham, DH1 3LE UK; 2 Laboratorio de Canales Iónicos, Instituto Multidisciplinario de Salud, Tecnología Y Desarrollo (IMSaTeD, CONICET-UNSE), Villa El Zanjón, 4206 Santiago del Estero, Argentina

**Keywords:** Electronic properties and materials, Electrical and electronic engineering

## Abstract

We report on the electrical behaviour of thin films of bovine brain microtubules (MTs). For samples in both their dried and hydrated states, the measured currents reveal a power law dependence on the applied DC voltage. We attribute this to the injection of space-charge from the metallic electrode(s). The MTs are thought to form a complex electrical network, which can be manipulated with an applied voltage. This feature has been exploited to undertake some experiments on the use of the MT mesh as a medium for computation. We show that it is possible to evolve MT films into binary classifiers following an *evolution in materio* approach. The accuracy of the system is, on average, similar to that of early carbon nanotube classifiers developed using the same methodology.

## Introduction

**Figure 1:**
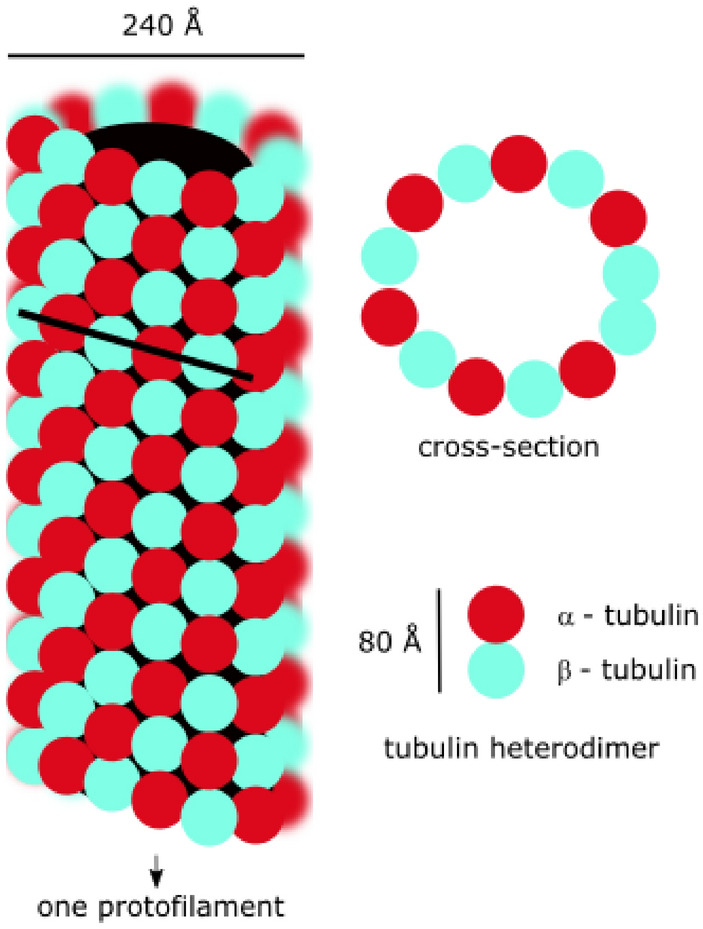
Structure of microtubule.

Microtubules (MTs) are polymers of the protein tubulin that form part of the cytoskeleton^1,2^. The MTs typically consist of 13 linear rows of tubulin dimers, known as protofilaments, which together form long hollow pseudo-helical cylinders with internal and external diameters of about 16 nm and 26 nm, respectively, depending on the precise number of protofilaments, and lengths commonly in the range 5–10 µm. Each protofilament within the MT comprises end-to-end negatively charged α- and β-tubulin dimers, consisting of amino acid residues with a net negative charge of about 20–30 electrons per tubulin, as depicted in Fig. 1. The negative charges, which are mainly distributed on the outer surfaces of the MT walls, will be balanced by positive ions in solution, leading to the formation of a double layer. A strong electric dipole is present along the MT axis and this can be aligned by an applied electric field^3^. Although the structural functions of MTs in some physiological processes have been widely accepted (e.g. cell mitosis, cell motility and motor protein transport), their precise role in others (e.g. neuron activity) remains elusive.

Some intriguing experimental data have been published on the electrical behaviour of MTs. For example, it has been demonstrated that MTs can work as biomolecular transistors capable of amplifying electrical information, as well as functioning as nonlinear electrical transmission lines that generate spontaneous electrical oscillations by changes in electric field and/or ionic gradients^4,5^. The voltage-dependent electrical resistance observed for MTs also suggests that these resemble memristor devices, offering potential for use as memory structures^6^.

In previous work, we have reported on the electrical behaviour of thin films of single walled carbon nanotubes (SWCNTs), which could be considered as simpler, non-biological versions of MTs^7,8^. By application of electric fields to a composite thin film of SWCNTs and liquid crystal (LC) molecules, we were able to bring the material to a state (i.e. change its morphology) where it was able to perform meaningful computation^9,10^. The methodology is known as *evolution in materio* (EiM)^11^ and offers an alternative to the conventional method of manufacturing electronic systems by which discrete components (resistors, capacitors, transistors) are linked together in the form of an integrated circuit.

Theoretical models for MT information processing have been reviewed in 2002^12^. In the same paper, it was suggested that MTs might even form a basis for quantum computation, with individual tubulins acting as qubits. Despite this and the other ideas noted above, there is a dearth of fundamental experimental data on the electrical properties of MTs in either their natural or dehydrated states. Here, we report an investigation into the DC electrical behaviour of MTs, together with some preliminary evidence on their ability to act as a medium for computation.

## Results and discussion

### MT morphology

**Figure 2:**
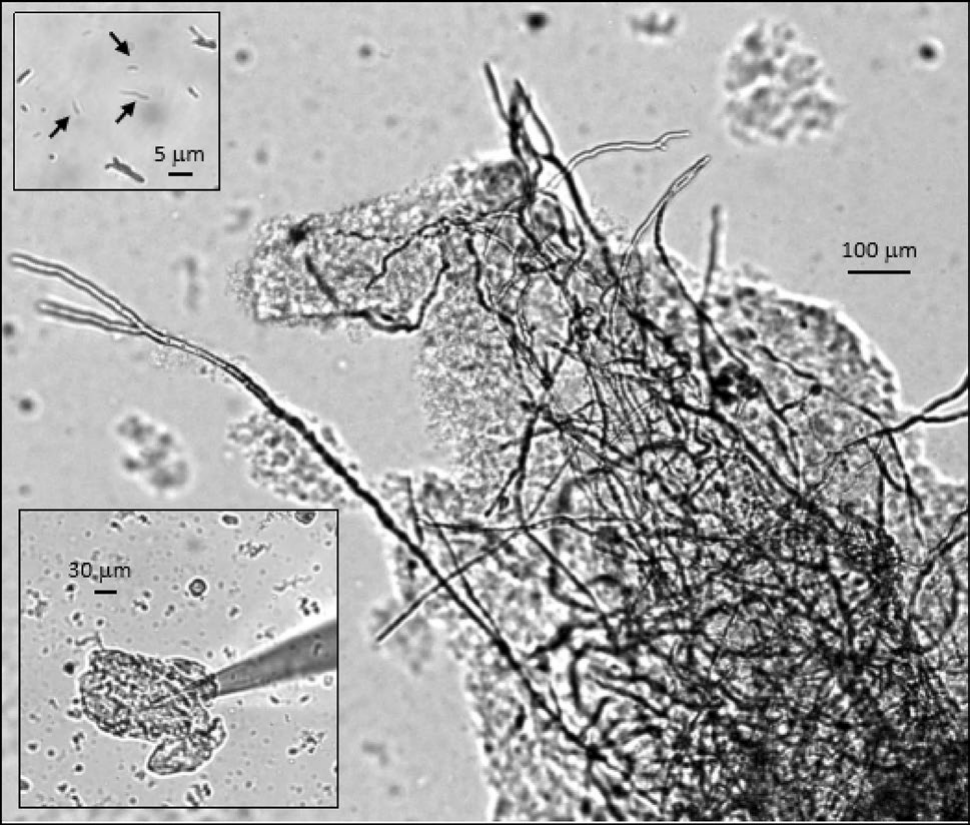
Optical microscope images of brain microtubule material used in the studies, including sheets, bundles and isolated MTs (×10, ×20, ×60), respectively. Upper left inset shows isolated MTs (arrows), as well as small macrotubes (e.g. top right). Lower left shows an MT sheet with apposed patch-clamp pipette.

Figure 2 shows typical optical microscope images of the MTs used in this investigation, revealing sheets, bundles and individual tubes. Calculations, given in Supplementary Information Note N1, suggest that these MT structures are more than 50 µm long, and are thereby able to bridge the gaps between the electrodes used in our electrical investigations.

### Patch clamp results

**Figure 3:**
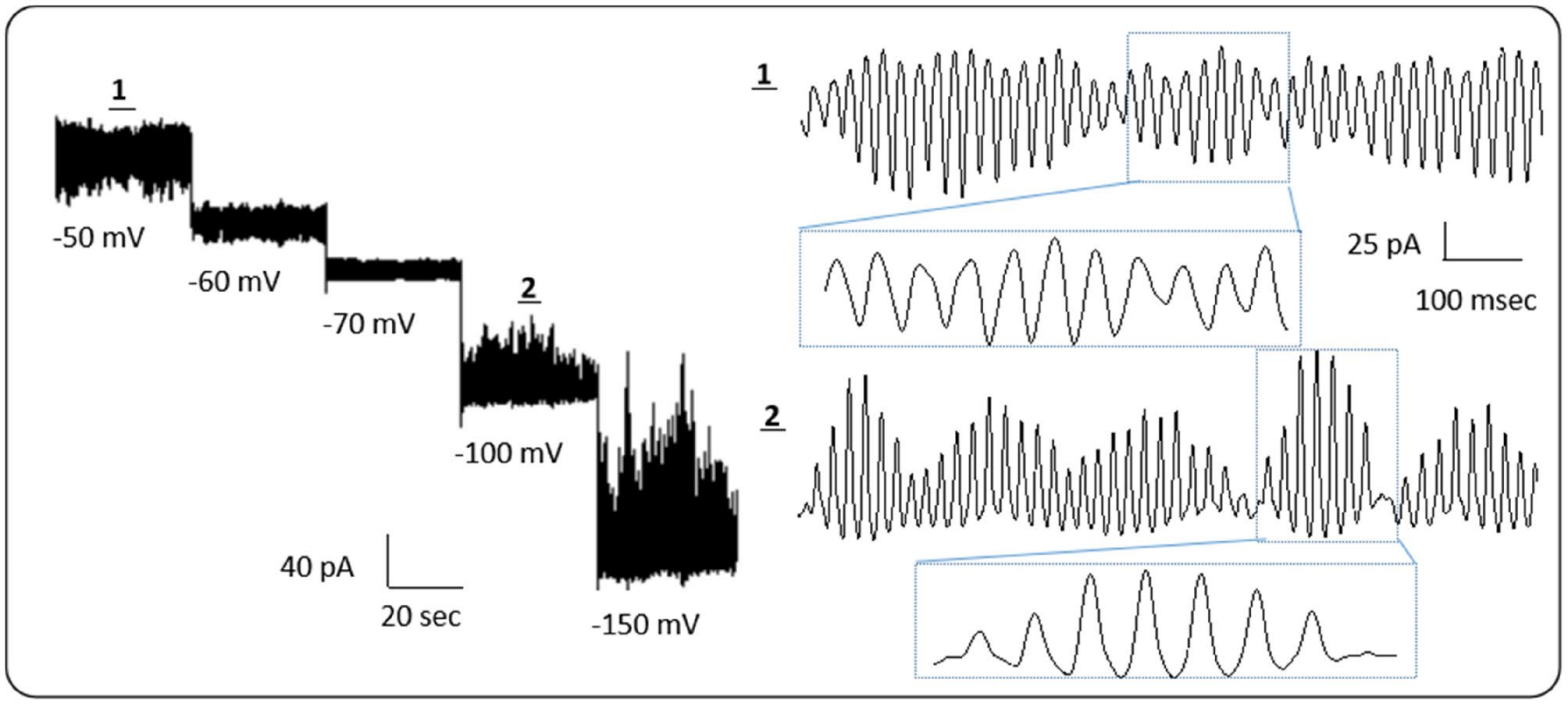
Current oscillations from a flat sheet of MTs in the presence of KCl 140 mM in the pipette and buffer A in the bath. Measurements at several holding potentials as indicated (left). Regions 1 and 2 are expanded on the right to reveal the presence of electrical oscillations. Data representative of four experiments.

The MT sheet was identified and voltage clamped as indicated^6^. Representative results from four experiments are shown in Fig. 3. Current oscillations were observed as previously described^5,13^. Non-oscillating MT sheets have also been reported^6^, showing memristive device properties. The same sample was dried on the coverslip in contact with air at room temperature. Thirty days after drying, the sample was rehydrated with distilled water and MT sheets were again voltage clamped. Current oscillations were again observed in the presence of KCl 140 mM in the pipette, and buffer A in the bath.

It should be noted that the current oscillations are generated without any external stimulation and possess a prominent fundamental frequency of 39 Hz. On average, the oscillations represent a 640% change in the conductance of the MT structures^5^. The origin of this electrical phenomenon is, at present, unclear. Similar oscillatory behaviour has been reported for frog hair cells^14^, which are thought to arise through the interactions of two hyperpolarisation ionic currents in the cell membrane. The electrical conductivity of MTs may possess both electronic and ionic contributions, and, therefore, the origin of these oscillations may be more complex. For example, spontaneous oscillations in electronic solid state (e.g. semiconductor) devices usually require the presence of negative differential resistance. The MT oscillations may play keys roles in the cell functions, such as providing a signalling mechanism to other cells, not only facilitating the transfer of electrical information in neurons, but also in controlling cell division and the transport of cargo in MT-driven organelles such as axons, cilia and flagella^5.^

### DC conductivity of dry MTs

**Figure 4:**
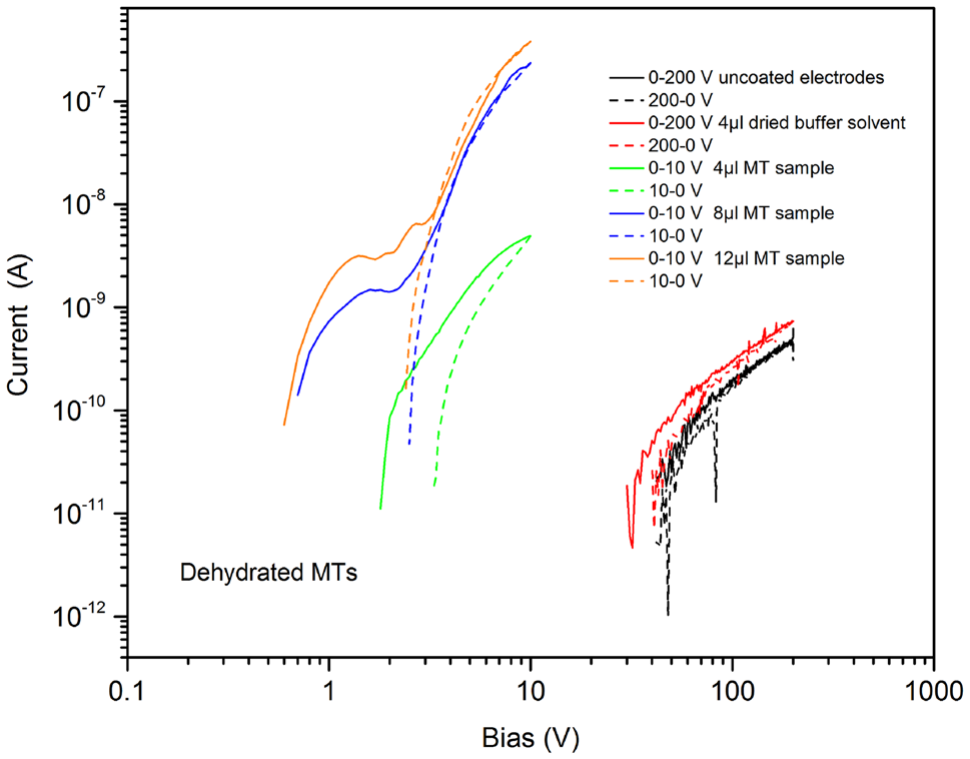
Current versus voltage curves for dried MT samples (formed from different amounts of MT solution). Reference data are shown for both uncoated electrode arrays and dried samples of the buffer solvent only. Measurements taken at room temperature and for both increasing (solid lines) and decreasing (dashed lines) applied voltages.

DC conductivity measurements were undertaken with MTs deposited onto patterned electrode arrays, as described in the Sect. 3. Ten sets of samples, each with 16 independent electrodes, were studied: these included reference specimens and samples with differing volumes of MT solution. The DC conductivities of the dried MT samples were found to increase with the volume of the MT solution that was initially deposited (12 µl > 8 µl > 4 µl > reference). Typical results for adjacent electrode pairs are shown in Fig. 4, for both an increasing and decreasing applied voltage. These data suggest that the measured currents are directly related to the presence of the MTs (in the case of the 4 µl sample, the current exceeded that for the reference device by at least three orders of magnitude). However, we also note that the dependence of the conductivity of the dried MT network on the volume of solution deposited is not linear, as the conductivity increases by roughly two orders of magnitude from 4 to 8 µl, and by a factor of less than two when the volume is further increased from 8 to 12 µl. These data indicate that the MT network crosses the percolation threshold at around 4 µl of deposited solution, and that thereafter, additional solution (MTs) only makes small changes to the electrical properties of the film.

As expected, from the symmetry of the electrode arrangement, similar results were obtained if the polarity of the applied bias was reversed. For an applied voltage of 10 V, maximum currents of 100s nA were observed. By contrast, the base conductivity of the glass slide and of the dried solvent without the MTs was very low (for 100 V, currents of 0.1 nA for uncoated glass and 0.2 nA for dried solvent), with the current approximately proportional to the applied voltage.

The current (*I*) versus voltage (*V*) behaviour for the MTs reveals hysteresis, time effects and current plateaus or peaks for some of the samples. Evidently, the conductivity mechanism for the MT samples is not a simple ionic process as dried samples of the solvent used to disperse the MTs (MES/EGTA/GTP/MgCl_2_) are insulating. Electronic conductivity might result from charge injected at the metallic electrode(s) and subsequent hopping or tunnelling via trapping sites associated with the highly polarisable aromatic rings of particular amino acids, such as tyrosine, tryptophan, phenylalanine and histidine. Tryptophan is the most polarisable amino acid^15^, possessing a particular double ring (an ‘indole ring’)—a six-carbon ring joined to a five-carbon ring with one nitrogen and four carbons. The distance between the tryptophan residues in MTs is around 2 nm, a spacing that could facilitate electron hops^12^.

For applied voltages greater than a few volts, the data for the MT samples shown in Fig. 4 reveal a power law dependence on *V*, with an exponent value of approximately five for *V* > 2 V, and hysteresis suggestive of charge-injection and/or trapping. In the case of an exponential trap distribution (with respect to electron energy), the space-charge limited current (SCLC) in a solid follows an *I*–*V* relationship of the form^16^:

1 $$I \propto d\left( {\frac{V}{{d^{2} }}} \right)^{m}$$

where *d* is the electrode separation and *m* is a constant, greater than unity, related to the trap distribution. Such characteristics are often observed in thin films of semiconductive polymers, such as poly(phenylene vinylene)^17^.

A current plateau or peak was sometimes observed in the *I*–*V* characteristics for increasing applied voltages, as evident for an applied voltage of approximately 1 V in the curves for the 8 µl and 12 µl samples in Fig. 4 (voltage sweep rate = 0.1 V s^−1^). This feature was reversible, so long as the applied voltage remained less than 10 V, and the current plateau would re-reappear on subsequent voltage scans. A plausible explanation is that this corresponds to the release of trapped electronic charge in the dehydrated MT film (the double plateau noted for the 12 µl sample suggest two types of trap). The current saturates with increasing bias as the traps empty; the injected electronic current then dominates with further bias increases. As the applied bias is reduced, the *I*−*V* characteristic follows the SCLC (power law) model. A variation on this hypothesis is that the current plateau is the result of ion movement. The ionic current saturates with increasing bias as (positive) ions accumulate at the (negative) electrode; the electronic current then dominates with further bias increase. As the applied voltage is reduced, only the electronic contribution to the conductivity is observed. On removal of the applied voltage, the ions diffuse from the electrode(s) and the current plateau is observed on re-measuring the conductivity.

The current plateau may be interpreted as a memory effect—it is present on the scan with increasing voltage, but not when the voltage decreases. Similar effects have been observed for a variety of (inorganic and organic) materials^16^. Further experiments should elucidate the conditions for switching between the memory states and determine if these could be exploited in a memory device. We have also recently reported nonlinear current voltage data for hydrated MTs, indicative of memory or memristor behaviour^6^.

**Figure 5:**
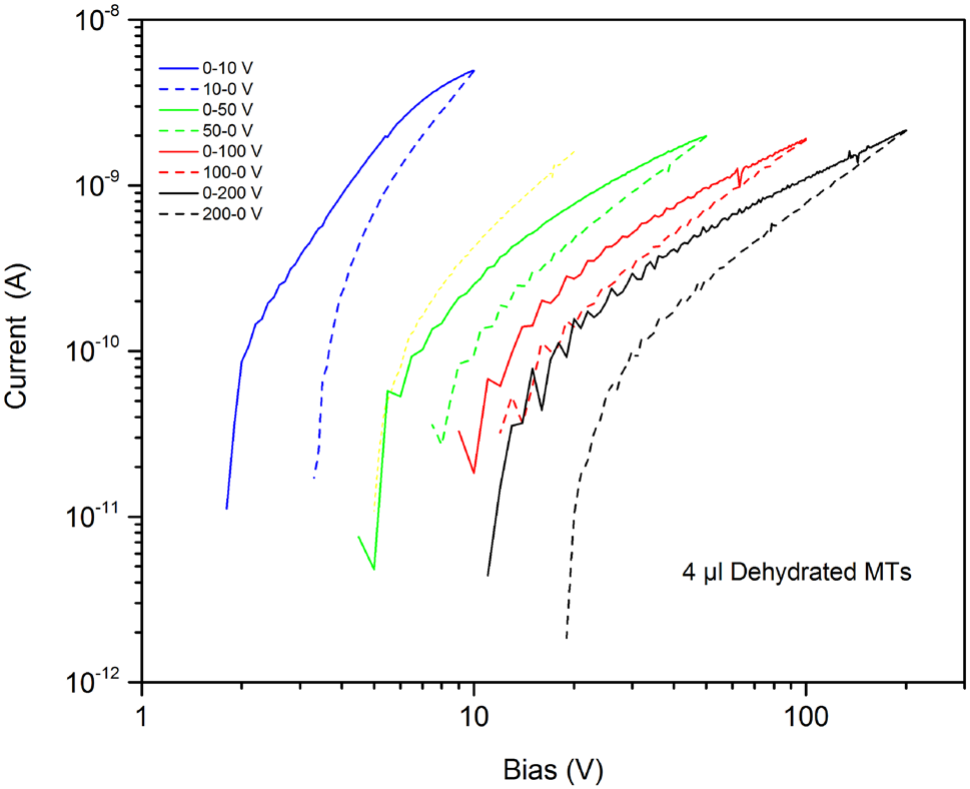
A series of consecutive current versus voltage measurements to progressively higher voltages for a dry 4 µl MT sample measured to progressively higher voltages. Measurements taken at room temperature and for both increasing (solid lines) and decreasing (dashed lines) applied voltages.

We emphasise that high applied voltages (> 10 V), the currents reduce with repeated bias sweeps, sometimes to the reference conductivity level for uncoated samples, as shown in Fig. 5 for a series of consecutive voltage sweeps to progressively higher voltages (the current data were recorded for both increasing and decreasing voltages in one sweep). These electrical data were irreversible. In this respect, it is important to appreciate that our electrode configuration will give rise to significant electric fields. For example, 100 V applied across 100 µm corresponds to a field of 10^6^ V m^−1^. Moreover, two-dimensional structural intermediates in MT assembly may present inter-protofilament distances in the order of less than 10 nm, rendering strengths of electric fields two orders of magnitude higher^18^.

These fields are likely to result in relatively high-power dissipations in the thin film MT mesh, producing permanent changes to its electrical connectivity in a similar way to the variations noted for carbon nanotubes in composite thin films^19^. In the case of rehydrated samples, the excessive heat is also likely to lead to evaporation of water.

The shapes of the current versus voltage characteristics are similar as progressively higher voltages are applied. The curves all exhibit the power law dependence of current upon voltage given by Eq. (1). However, the exponent *m* decreases towards unity (i.e. Ohmic conductivity) for higher applied voltages, indicating that the injection of charge from the electrode(s) is becoming more difficult. It is therefore likely that electrophoretic effects or high-power dissipation in dry MTs disrupt their connectivity (this is suggested by optical microscope studies of our MT samples following the application of a large voltage and is discussed later)^20^.

Stability tests, in which a constant voltage was applied to the MT thin film for approximately one hour, also revealed a decrease in the current. This effect was dependent on the magnitude of the applied voltage; the greater the voltage applied, the more rapidly the current decreased. Occasionally, the current *increased* to a maximum before it started to decrease (see later results for hydrated MTs), which may be evidence of an evolving MT electrical network over time.

### DC conductivity of hydrated MTs

The reference uncoated electrodes showed an increase in conductivity when deionised water was added (currents up to 10 nA, with *V* = 10 V), which reduced over time to the initial (dry) level. This behaviour reflects the residual conductivity of the deionised water (conductivity 5 µS m^−1^).

Electrodes coated with the reference buffer solvent without MTs exhibited a larger conductivity when water was added (currents up to 10 µA). The conductivity then decreased to a base level after approximately 100 h. Again, the explanation is that this observation results from ionic conductivity as the dried solvent will contain many ionic species, which will be activated on hydration. The currents showed various peaks/instabilities and were independent of voltage for applied biases greater than about 10 below V. Figure S1 in the Supplementary Information is an example of the current versus voltage behaviour for a 4 µl solvent-only reference sample before, immediately following hydration and after 114 h.

**Figure 6:**
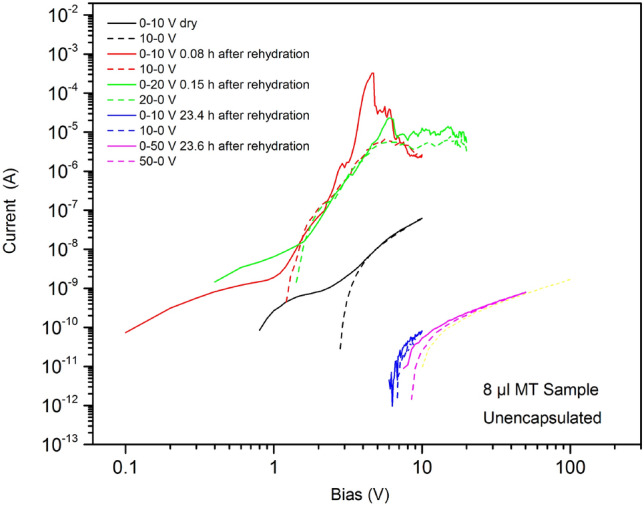
Conductivity for 8 µl unencapsulated MT sample before and at various times following hydration measurements taken at room temperature and for both increasing (solid lines) and decreasing (dashed lines) applied voltages.

The conductivity of the dry MT samples also increased with added water, but to much higher levels compared to the reference samples, showing that MTs in solution provide additional conductivity over rehydrated solvent. Figure 6 shows typical data for an 8 µl MT sample.

The *I*–*V* curves reveal a power law dependence of *I* on *V* (exponent > 5) below 10 V, similar to that observed for the dehydrated MT samples (Fig. 4), but with several peaks in the measured current. The currents noted for the reference saline samples were of the order of microamps, whereas milliamp currents could be achieved for the MT samples, with only a few volts applied. We therefore suggest the same electronic conductivity process present in the dry MT samples dominate in the hydrated MT samples. We propose that the wet conditions simply allow a more extensive (and, probably, anisotropic) MT network to form, with an enhanced conductivity over the dry MT film. We have observed similar effects for SWCNTs dispersed in liquid crystals^19^. A significant conductivity increase might be expected if much of the MT network was rearranged in a favourable manner, e.g. in the form of bundles/wires and close to the percolation threshold. The reproducibility of the highly conductive hydrated samples was found to be poorer than that of the dried samples. We suggest this was related to the higher power dissipation in the thin film (approaching mW for a few applied volts) which could lead to disruption of the MT electrical network.

The conductivity of the MT network decreased during the experiments, as shown in Fig. 6. We ascribe this to drying of the film as the conductivities could be recovered by rehydration. The low conductivity state at long times shown in Fig. 6 represents the dried film, however, we note that the conductivity observed is lower than that of the dried samples shown in Fig. 4 for equivalent MT solution volumes. The difference between these data is that the film in Fig. 6 is dried during an applied bias stress, whilst in Fig. 4 the film is dried without applied bias. Supplementary Information, Figure S2, shows that MT films dried under the presence of an electric field have a region devoid of MTs surrounding one of the electrodes (usually the negative electrode) and the presence of globules of material (denatured MTs) on and between some of the electrode tracks. Both phenomena are probably related to an electrophoretic process^20^ and/or excessive heat generated within the conductive MT network (e.g. resulting from the relatively high levels of injected currents). The impact of water evaporation and thermal stresses limited our studies to low applied voltages (a few volts) and to relatively short experimental times.

The above results are consistent with the expected properties of the proteins in solution, which are physiologically not exposed to extremely large electric fields. In cellular context(s) with resting potentials in the order of 0.1 V, biological membranes (and thus proteins apposed to them) may be subject to electric fields in the order of 10^6^ V m^−1^. Under these conditions voltages much larger than 10 V may exert effects inconsistent with their biological properties. Nonetheless, it is interesting to observe that MTs are exposed to stabilizing mechanisms, including direct interactions with heat shock proteins such as Hsp90^21^, a very abundant cellular protein that counters the mechanical lability of isolated tubulin oligomers in solution. A recent study^22^ indicated that the strength of protofilament interactions in 2D sheets prior to MT formation are comparable to the strength of the curvature energy. Thus, the shape of the free energy landscape appears to be crucial in explaining the mechanism of MT shrinkage, where the unzippered protofilaments will fluctuate in a set of partially peeled off states where subunit dissociation will reduce their length. It is worth mentioning that MT meshes as shown in Fig. 2, and electrically assessed as voltage-clamped 2D MT sheets, are highly stable in regard to changes in pH, ionic composition, and temperature (see Figure S3 in Supplementary Information).

For some hydration experiments the measured current increased over many minutes before decreasing to a low level, which often occurred for the second and subsequent hydration cycles. An example is provided in the Supplementary Information, Figure S4. Noisy electrical behaviour was observed prior to the decrease in the current. We reason that these current fluctuations are not related to the 39 Hz spontaneous electrical behaviour observed in MTs^13^ as our DC data are recorded every 10 s. Experiments using hydrated samples sealed with glass cover slips revealed that it was possible to retain high conductivity levels in the hydrated MT samples for a few days.

### Evolutionary computation

**Figure 7:**
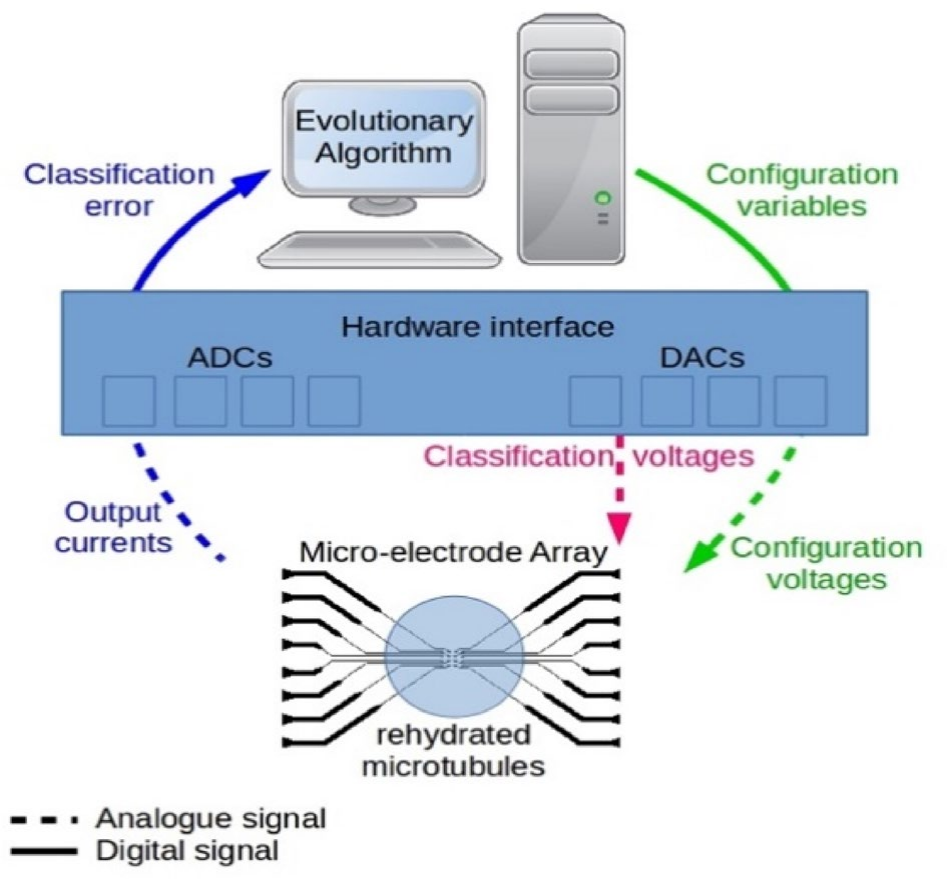
Schematic diagram of an EiM processor described in the text.

A schematic diagram of our EiM processor is shown in Fig. 7. The morphology of the MT network will determine its initial electrical behaviour. ‘Programming’ is then achieved by an evolutionary algorithm (EA) that adjusts the MTs’ configuration for a target application. The EA assesses the suitability of a population of candidate configurations for the task at hand, in this case a two-attribute classification problem. Each configuration represents a particular set of connections to the MT network and configuration voltage levels. Our experimental arrangement allows input and configuration voltages to be applied to the MTs, and output currents to be measured.

The conductivity of the dehydrated samples was too low for our hardware interface. Our investigations therefore focused on the more conductive hydrated MT samples, both air-exposed and encapsulated. The population average and the minimum classification errors obtained with a set of reference (uncoated) electrodes remained around 50% during training and verification, demonstrating that the system is unable to provide solutions to the classification problem better than a random guess if MTs are not deposited. This was consistent across all of our experiments. Further, the EA was unable to solve the classification problem in an open-circuit configuration, when only noise was picked up by the microcontroller.

Turning to the performance of the EiM system when MTs were deposited on the electrodes, Figure S5 of the Supplementary information shows that both the population average and the minimum classification errors obtained with a 12 μl MT rehydrated film during training were lower—at 26%—than those obtained with the uncoated electrodes. Furthermore, the verification error obtained in this instance was approximately 27%, which means that the solution evolved during training was capable of generalising to a new set of data. These results are not optimal but are in the range of those obtained in experiments undertaken with SWCNT/LC composites previously reported^23^. On average, the optimum training and verification errors for the MTs were 24% and 26%, respectively. This suggests that the material has the potential to be evolved into a classifier following an EiM approach. However, results varied across experiments and in the majority of cases, the verification error differed from the training error by more than 20%.

**Figure 8:**
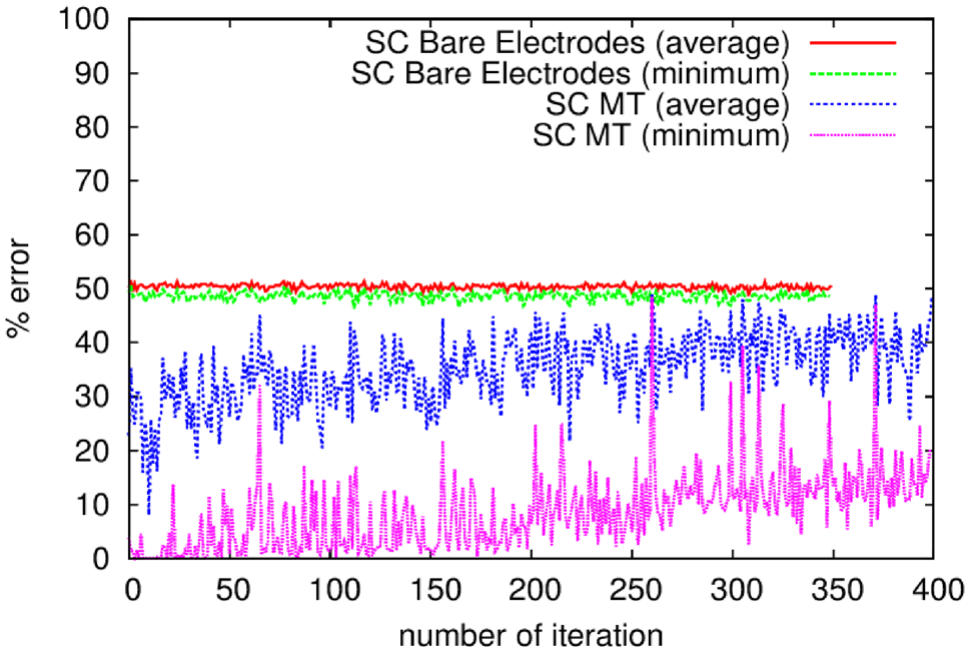
Convergence of the percentage error for an uncoated electrode array and a sealed, rehydrated 12 µl MT film.

Improved training results were achieved using MT samples that were sealed to slow the process of water evaporation, as shown in Fig. 8. The changing percentage error with iteration number is due to the evolving configuration (i.e. configuration voltages) on the one hand, and evolving material properties on the other (i.e. the drying and changing conductivity of the film). The average and minimum classification errors obtained with the sealed, 12 μl MT rehydrated film were lower than those obtained with unsealed MTs, achieving a minimum 0% error for the example shown. This suggests that the algorithm was able to configure the MT film such that all instances from the training dataset were classified correctly. We also observe that the minimum training error drops to less than 5% in the first iteration, and reaches a minimum in less than ten generations, thus comparing favourably with our previous work involving carbon nanotubes^10^. However, Fig. 8 also reveals that the error increases with the number of iterations, to reach final values of 20% minimum error and 40% average error when the training terminates. The verification error matches these final values.

We note that whilst a large verification error may indicate that a trained classifier does not generalise to unseen data well, we suggest that in this case the large verification error is at least in part due to irreversible changes in network conductivity. Our reasoning for this is that each training iteration took 30 s, and as such, Fig. 8 represents a period of 3 h and 20 min during which sufficient bias is applied to cause irreversible changes in the sample. Further, a gradual increase in average training error is observed beyond approximately 10 iterations, something which has not been observed in other EiM systems, and thus may indicate deterioration (or at least a change) in the morphological network.

This supposition is consistent with the measured currents decreasing with time, even though the voltage levels applied to our samples remained between 0.5 and 3.5 V throughout training. Once the network conductivity fell below a certain value, it would act as an open circuit and the error would approach 50%, as found for the reference samples. Furthermore, examples of the morphological effects shown in Fig. S2 were observed in some of our samples following the training procedure. Although the DC conductivity data discussed in our previous section indicated that the sealed, hydrated MT samples are reasonably reproducible over a period of several days, it seems that the application of multiple voltages during the training process of several hours can degrade the electrical network in our samples more rapidly. Our further work is therefore focused on improving the sensitivity of our hardware/materials interface to enable much lower currents to be measured.

## Methods

### Materials

The bovine brain MTs were obtained in Buenos Aires, Argentina. Patch-clamp measurements were undertaken in the same laboratory, as previously reported^13^. The MT samples were deposited onto electrode arrays, provided by Durham. These were subsequently dried and shipped to the UK for DC electrical and computational experiments.

The MTs were isolated from bovine brain by cycles of polymerisation and depolymerisation as originally described by Ávila et al.^24^, with modifications described by Cantero et al.^13^. Briefly, fresh bovine brains were obtained from a local slaughterhouse, and immediately processed. The brains were rinsed with cold PBS buffer (×3) and maintained at 4 °C throughout the procedure. These were then chopped and added to 1 ml of isotonic buffer per gram of tissue containing: phenylmethylsulfonyl fluoride (PMSF) 1 mM, aprotinin 1 μM, leupeptin 1 μM, and pepstatin 1 μM (MP Biomedicals LLC, Santa Ana, CA, USA). The tissue was homogenised with a blender at low velocity and a Potter homogeniser. The homogenate was centrifuged for 30 min. at 25,000*g* (×2). The supernatant was diluted in an electrolyte buffer solution containing, in mM: 2-(*N*-morpholino)ethanesulfonic acid (MES) 100 at pH 6.7, ethylene glycol-bis(β-aminoethyl ether)-N,N,N′,N′-tetraacetic acid (EGTA) 2.0, MgCl_2_ 1.0, PMSF 1.0, guanosine-5′-triphosphate (GTP) 1.0, and glycerol 30% (v/v)—Buffer A. The mixture was ultra-centrifuged for 45 min at 100,000*g* at 25 °C. Besides isolated MTs, large two-dimensional MT sheets were easily identified under DIC and immunochemistry (anti-α tubulin antibody, Santa Cruz Biotechnol, Dallas, TX, USA) with an Olympus IX71 fluorescence inverted microscope^13^. Samples were kept frozen at −20 °C until further use. This ‘original sample’ was dehydrated for a month and rehydrated with distilled water.

### Electrophysiological data acquisition

Electrical recordings from voltage-clamped MT sheets were undertaken using the original sample and after rehydration. The electronic setup included a conventional patch clamping amplifier (Axopatch 200B, Molecular Devices, Sunnyvale, CA, USA), directly connected to the sample. The experimental procedures are summarised in the Supplementary Information, Figure S6. Please note that the electrical properties of oscillating^13^ and non-oscillating^6^ MT sheets have been addressed extensively in previous reports.

### DC electrical conductivity measurements

The DC conductivity measurements were undertaken with MTs deposited, by drop casting, onto patterned electrode arrays. These were fabricated using conventional etch-back photolithographic techniques from chromium gold on borosilicate glass slides. Grid arrays were prepared on each slide; the contact pads had a diameter of 50 µm and a 100 µm pitch (distance between electrode centres)^9^. A washer was attached to the slides surrounding the electrodes to contain a liquid. A photograph showing the configuration of the electrode arrays (uncoated and without the washer) on a glass slide is provided in the Supplementary Information, Figure S7. The slides were coated with different MT volumes (4 µl, 8 µl and 12 µl) in Buffer A and allowed to dry. Photographs of the reference electrodes (no MTs) and those coated with MTs are shown in the Supplementary Information, Figure S8.

DC electrical measurements were performed using a Keithley 2635A sourcemeter with a custom designed MATLAB interface program. A simple encapsulation scheme, using a glass cover slip fixed in place with a two-component epoxy adhesive (araldite), was adopted in some experiments. However, unless stated, all measurements were made at room temperature with unencapsulated and air-exposed samples. The voltage scan rates were as follows: 0–10 V–0.1 V s^-1^; 0–20 V–0.2 V s^-1^; 0.50 V–0.5 V s^-1^; 0–100 V and 0–200 V–1 V s^-1^.

### Computing problem formulation

The ability of EiM to evolve a computational response in MTs was first assessed as proof-of-concept using a binary, two-dimensional, classification problem defined in our previous work^10,23,25^. The experimental protocol is outlined in Note N2 and Figure S9 in the Supplementary Information.

## Supplementary Information

Supplementary Information.
