# Supplementary material for: Electrical behaviour and evolutionary computation in thin films of bovine brain microtubules
Source: Sci Rep. 2021 May 24;11:10776. doi: 10.1038/s41598-021-90260-0 (PMC8144580; doi:10.1038/s41598-021-90260-0)
Supplement: Supplementary file 1 — Supplementary Information. [file 41598_2021_90260_MOESM1_ESM.docx]

**Electrical Behaviour and Evolutionary Computation in Thin Films of Bovine Brain Microtubules**

Eléonore Vissol-Gaudin,^1^ Chris Pearson,^1^ Chris Groves,^1^ Dagou A. Zeze,^1^

Horacio F. Cantiello,^2^ María del Rocío Cantero^2^

and Michael C. Petty^1^

^1^Department of Engineering, Durham University, South Road, Durham DH1 3LE, United Kingdom

^2^Laboratorio de Canales Iónicos, Instituto Multidisciplinario de Salud, Tecnología y Desarrollo (IMSaTeD, CONICET-UNSE), Villa El Zanjón, Santiago del Estero, 4206 Argentina

Correspondence should be addressed to M.C.P. (email: m.c.petty@durham.ac.uk)

**Supplementary Information**

**List of Figures**

**Fig. S1: Conductivity for reference (no MT) sample on hydration**

**Fig. S2: Morphological changes following extended measurements**

**Fig. S3: Effects of environmental changes on MTs sheet oscillations**

**Fig. S4: Current versus time for MT sample following rehydration**

**Fig. S5: Convergence of error function for unencapsulated 12 µL MT film**

**Fig. S6: Patch-clamp experimental procedures**

**Fig. S7: Optical microscope images of uncoated electrode arrays**

**Fig. S8: Optical microscope images of electrode arrays**

**Fig. S9: Training and verification datasets**

**List of Notes**

**Note N1: Quantification of MTs**

**Note N2: Background to computational experiments**





Figure S1: Conductivity for 4 µl reference solvent-only (no MTs) sample before (dry) and after rehydration. Measurements taken at room temperature and for both increasing (solid lines) and decreasing (dashed lines) applied voltages.


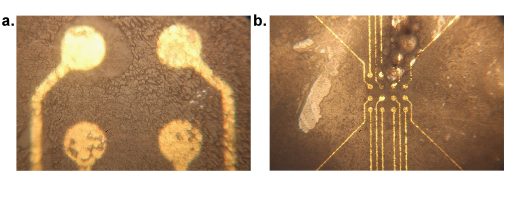


Figure S2: Optical microscopy images for MT samples following hydration and drying cycles. The 'halo' surrounding one of the electrodes, such as that shown in **a**. was frequently observed in MT films following the application of a large (> 10 V) voltage. This was usually the negative electrode. These samples possessed very low conductivity, similar to that of the glass substrate, after the measurements. **b.** The presence of globules of material on and between some of the electrode tracks following extended measurements (e.g., hydration, drying, rehydration, drying) is apparent with large applied voltages. We suggest that these are denatured MTs.

Figure S3: Effects of environmental changes on MTs sheet oscillations. Neither high CaCl_2_ (a) nor low pH (b) modify electrical oscillations. However, the magnitude of the signals (but not the frequency, not shown) is temperature dependent (c). In all, electrical oscillations of flat sheets of MTs are highly stable within the physiological range.

**

**

Figure S4: Current versus time data, measured at room temperature, for the second rehydration of a 8 μl MT sample. For a 10 V fixed applied voltage, the measured current first increases, over a period of approximately 30 min, before decreasing rapidly afterwards. The noisy nature observed for the current probably reflects a changing MT electrical network over time, i.e. connections between the MTs are formed by movement and broken with excessive heat dissipation


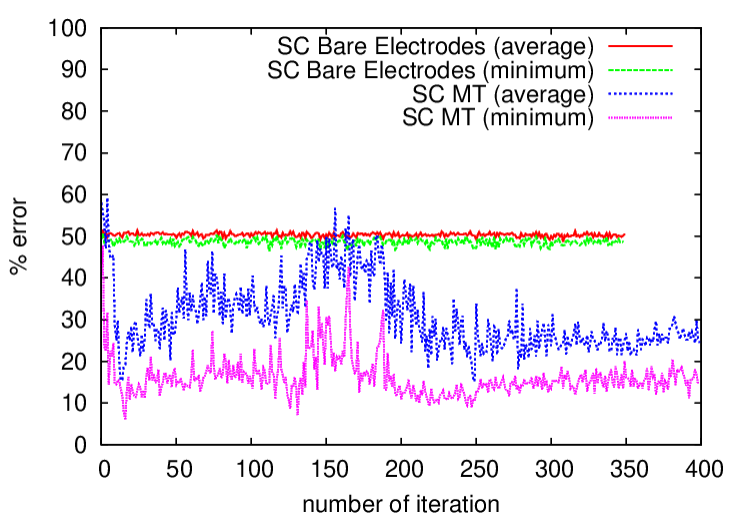


Figure S5: Convergence of the percentage error for an array of uncoated (bare) electrodes (reference device) and a rehydrated 12 µl MT film. The population average and the minimum classification errors obtained with a set of uncoated electrodes remain around 50% during training and verification. This is consistent across experiments. The algorithm used in the training process is unable to solve the classification problem in an open circuit configuration, when only noise is being picked up by the microcontroller. The population average and the minimum classification errors obtained with the rehydrated 12 μl MT film during training are *lower* than those obtained with the uncoated electrodes, which suggests that the solution evolved during training was capable of generalising to a new set of data.


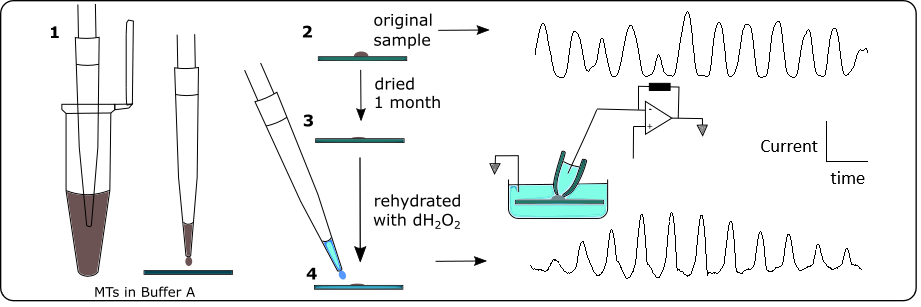


Figure S6: Schematic diagram of MT film formation and effect of hydration-dehydration cycles on patch-clamp measurements. Largely 2D-shaped MT sheets from both samples were identified in the preparation, approached by the patch pipette and sealed by light positive pressure of the tip onto the surface.^a^ Seal resistance, and thus the quality of the patch was obtained by imposing 1-5 mV square pulses. A decrease in current was a reflection of the increased resistance that reached GΩ values. The MT sheet electrical currents were followed by applying different voltage protocols driven from the head stage of the patch-clamp amplifier. Usually, the stability of the patch was such that long-lasting experimental conditions could be explored on the MT sheet. Patch pipettes were made from soda lime capillary tubes (Biocap, Buenos Aires, Argentina) with 1.25 mm internal diameter. Pipette tips were pulled with a pipette puller (PB-7, Narishige, Tokyo, Japan) and fire polished (MF-9, Narishige, Tokyo, Japan) to a tip diameter of 3-4 μm. Electrical signals were acquired and filtered at 10 kHz, digitised with an analogue-digital converter (Digidata 1440A, Molecular Devices) and stored in a personal computer with the software suite pCLAMP 10.0 (Molecular Devices), also used for data analysis. Sigmaplot Version 10.0 (Jandel Scientific, Corte Madera, CA) was used for statistical analysis and graphics. Electrical oscillations remain present after samples were kept dehydrated at room temperature for a month.


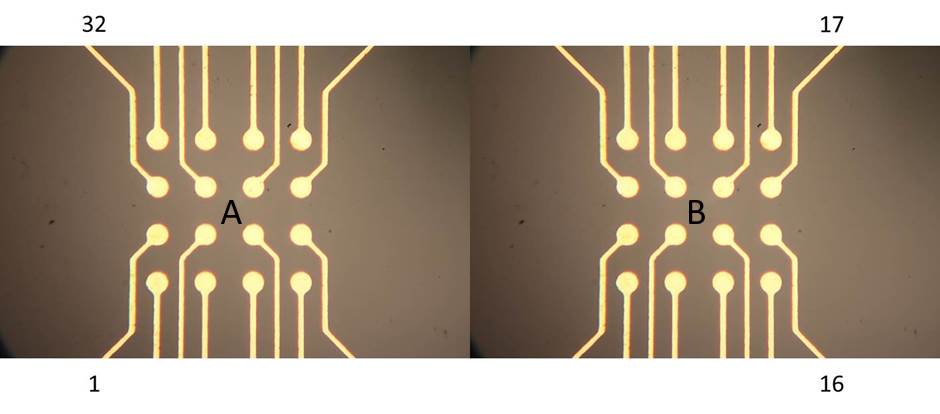


Figure S7: Electrode arrays A and B on a glass slide. Contact pad diameter 50 μm; electrode pitch 100 μm. Each slide comprised 16 individual electrodes. Generally, one set of electrodes (e.g. A) was used for MT deposition while the other (B) formed a reference (either uncoated or coated with the ionic electrolyte solution).


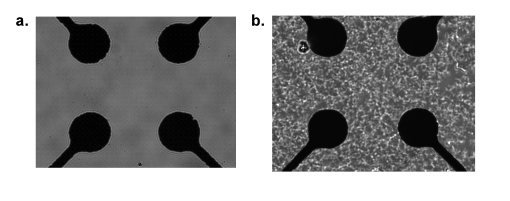


Figure S8: Optical microscope images for **a.** reference electrode set coated with 4 µl of the buffer solution and subsequently dried and **b.** electrode array following deposition and drying of 8 µl of the MT solution. There is clear evidence of MT deposition with a uniform film morphology between and around the individual electrodes.


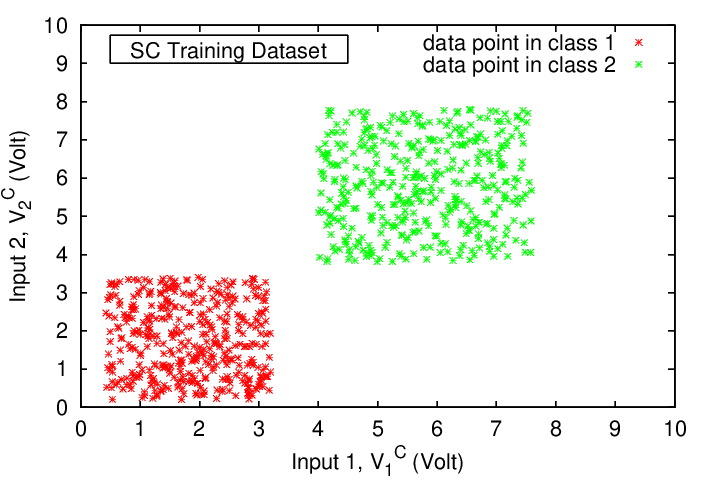

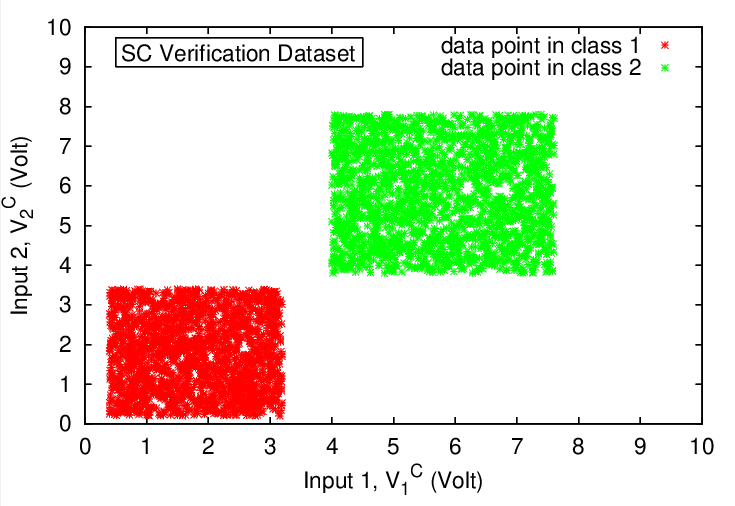


1. (b)

Figure S9: Distribution of (a) training and (b) verification datasets in the two-dimension (2D) input space, where each dimension denotes an attribute of the problem.

Note N1: Quantification of MT density in the experimental samples was as follows. The protein concentration in the original preparation was approximately 33 mg/mL, as determined by the BCA method. Therefore, there should be approximately 132 mg protein in a 4 mL sample. Because tubulins are ~50 kD proteins (8.3 x 10^-20^ g/monomer) one tubulin monomer would represent 8.3 x 10^-20^ g protein. Thus, there should be 132/8.3 x 10^-20^ = 1.6 x 10^21^ monomers in the sample. By assuming a 10 nm length per ring of 13 monomers (in the cylindrical shape of the MT, see Fig. 1, main text), a 1 µm long MT (1000 nm) would contain approximately 1.3 x 10^3^ monomers, meaning that the upper bound would be a total number of 10^19^ MTs in the 4 µl sample. Considering that both MAPs (microtubule-associated proteins) and other proteins will likely be associated with the MTs in the preparation, this figure could be reduced by as much as by half in the sample (5 x 10^18^ MTs/4 µl sample). It could therefore be considered that the preparation, as used, may contain MT macrotubes, bundles and sheets that will be longer than 50 µm (as shown in Fig. 2, main text), thereby bridging the gaps between the electrode sets.

Note N2: We refer to the dataset underpinning this problem as the SC (Separate Classes) dataset, in which each data point has a class that is determined by two attributes that take the form of voltages, *V*_1_*^C^* and *V*_2_*^C^*. The two classes are defined as follows:

$0.4V\leq{V_{1}}^{C}\leq3.2V$ and $0.2V\leq{V_{2}}^{C}\leq3.4V$: Class 1

$3.8V\leq{V_{1}}^{C}\leq7.5V$ and $4V\leq{V_{2}}^{C}\leq7.6V$: Class 2

The SC dataset comprises two parts: a training dataset shown in Fig. S9(a) containing 800 data points; and a verification dataset shown in Fig. S9(b), containing 4000 datapoints. The training dataset is used during the process of configuring the material, whilst the verification dataset is used to test how well the trained material classifies unseen data. In each case, attributes were generated randomly using a uniform distribution with class limits as defined above. The process of training the classifier, and subsequent verification of its operation, is provided in detail elsewhere^b^ and so will only be described briefly here. The first stage is to train the initially unconfigured material via an iterative process such that it adopts a state that can accurately classify the training data. This process is achieved by first generating a series of initial guesses at configurations (configuration voltages in this case), each of which constitutes a member of a population of possible solutions to the training problem. For each configuration applied to the material (and thus population member), the entire training dataset is sent sequentially in the form of applied voltages to the material, and output currents *I*_O1_ and *I*_O2_ are measured. The quality of solution each configuration, *c*, provides is quantified by comparing the inferred class at the output to the real class and noting the number of incorrect classifications, known as the error value. The interpretation scheme used to infer a class from the output currents is as follows:

*I*_O1_ / *I* _O2_ < *R*: Class 1

*I*_O1_ / *I* _O2_ > *R*: Class 2

where *R* here is a configuration variable optimised by the evolutionary algorithm. An evolutionary algorithm based on Differential Evolution selects, mutates and recombines members of the population so as to minimise the error value in successive generations.^c,d^ Once the members of the population to be carried forward into the next generation is determined, the evaluation of the population against the training data is repeated. This process continues until either the minimum error is reached, or the number of generations reaches 400. In either case, when training terminates, the best configuration (i.e. that producing minimum training error) is selected to be re-applied to the material along with the verification data. It must be noted that (i) the best configuration may belong to an early generation, i.e. the material state may have changed when it is re-applied and (ii) the verification data are statistically similar to the training data, but as yet unseen by the nanomaterial composite. The mis-classification error rate (expressed as a percentage) is known as the verification error. It provides a quantitative measure of the generalisation property of the evolved material / configuration voltage combination. In other words, the verification error is a measure of the MT classifier quality and can be used to compare different machine learning approaches to the same (SC) classification problem.

References

^a^Cantero, M.R., Perez, P.L., Smoler, M., Villa Etchegoyen, C. and Cantiello, H.F. Electrical oscillations in two-dimensional microtubular structures. *Sci. Rep*. **6**, 27143 (2016).

^b^Vissol-Gaudin, E., Kotsialos, A., Massey, M.K., Groves, C., Pearson, C., Zeze, D.A. and Petty, M.C. Solving binary classification problems with carbon nanotube/liquid crystal composites and evolutionary algorithms. In *2017 IEEE Congress on Evolutionary Computation (ICRC)*: 274-281 (2017).

^c^Storn, R. and Price, K. Differential evolution–a simple and efficient heuristic for global optimization over continuous spaces. *Journal of Global Optimization* **11**, 341-359 (1997).

^d^Pedersen, M.E.H. Good parameters for differential evolution. *Technical Report HL1002, Hvass Laboratories* (2010).
